# The degree of microbiome complexity influences the epithelial response to infection

**DOI:** 10.1186/1471-2164-10-380

**Published:** 2009-08-18

**Authors:** Jeffrey J Mans, Kate von Lackum, Cassandra Dorsey, Shaun Willis, Shannon M Wallet, Henry V Baker, Richard J Lamont, Martin Handfield

**Affiliations:** 1 Department of Oral Biology, College of Dentistry, University of Florida, Gainesville, Florida; 2 Department of Periodontology, College of Dentistry, University of Florida, Gainesville, Florida; 3 Department of Molecular Genetics and Microbiology, College of Medicine, University of Florida, Gainesville, Florida

## Abstract

**Background:**

The human microflora is known to be extremely complex, yet most pathogenesis research is conducted in mono-species models of infection. Consequently, it remains unclear whether the level of complexity of a host's indigenous flora can affect the virulence potential of pathogenic species. Furthermore, it remains unclear whether the colonization by commensal species affects a host cell's response to pathogenic species beyond the direct physical saturation of surface receptors, the sequestration of nutrients, the modulation of the physico-chemical environment in the oral cavity, or the production of bacteriocins. Using oral epithelial cells as a model, we hypothesized that the virulence of pathogenic species may vary depending on the complexity of the flora that interacts with host cells.

**Results:**

This is the first report that determines the global epithelial transcriptional response to co-culture with defined complex microbiota. In our model, human immortalized gingival keratinocytes (HIGK) were infected with mono- and mixed cultures of commensal and pathogenic species. The global transcriptional response of infected cells was validated and confirmed phenotypically. In our model, commensal species were able to modulate the expression of host genes with a broad diversity of physiological functions and antagonize the effect of pathogenic species at the cellular level. Unexpectedly, the inhibitory effect of commensal species was *not *correlated with its ability to inhibit adhesion or invasion by pathogenic species.

**Conclusion:**

Studying the global transcriptome of epithelial cells to single and complex microbial challenges offers clues towards a better understanding of how bacteria-bacteria interactions and bacteria-host interactions impact the overall host response. This work provides evidence that the degree of complexity of a mixed microbiota *does *influence the transcriptional response to infection of host epithelial cells, and challenges the current dogma regarding the *potential *versus the *actual *pathogenicity of bacterial species. These findings support the concept that members of the commensal oral flora have evolved cellular mechanisms that directly modulate the host cell's response to pathogenic species and dampen their relative pathogenicity.

## Background

The human microflora is an extremely complex ecosystem characterized by the simultaneous presence of a large number of "normal" colonizers, associated with health, and thriving in a dynamic environment alongside opportunistic and pathogenic species. Since health is the most common state of a host, it has been speculated that the autochthonous flora has co-evolved with its host to interact in a balanced state that is beneficial to both the host and the microflora. There are an appreciable number of benefits to the host that the indigenous flora is thought to provide, including the synthesis of vitamins (B complex and K), the prevention of infection by pathogens (by direct competition for niches or by immune cross-reactivity), and its impact in the normal development of the immune system [1,2]. Indeed, observed differences between pathogen-stimulated and commensal-stimulated immune responses are suggested to provide important insights toward understanding the molecular aspects of host-microbiota interactions [2-4]. Most notable examples include the intestinal commensals whose interactions with the gut epithelium trigger both innate and adaptive immune responses, influence epithelial cell proliferation [5], and induce commensal-specific IgA production and secretion in the gut to keep commensal species in check [6]. More recently, an oral commensal organism, *Streptococcus salivarius *strain K12, was demonstrated to antagonize *Pseudomonas aeruginosa*-induced IL-8 secretion from human bronchial epithelial cells, suggesting a role for commensal species in modulating human epithelial cell immune responses in the nasopharynx [4]. Likewise, a separate group has described the capability of *S. cristatus *and certain other streptococcal species to dampen the IL-8 response induced by infection with the periodontal pathogen *Fusobacterium nucleatum *in four different epithelial cell lines. These observations demonstrate that polymicrobial infection of epithelial cells with oral streptococcal species and commonly associating pathogens can attenuate the proinflammatory effects elicited during infection of these cells with the pathogens [7].

Since host and microbiota interactions are inherently unstable, disease may arise at the mucosal surface of a susceptible host when a perturbation occurs in the epithelial environment leading to "unintended" (in an evolutionary sense) consequences of immune or other host cell activity. Such instances include both hyper-acute immune responses as well as the converse situation, such as when the host becomes immuno-compromised. Further, the complex etiology of oral infectious diseases involves consortia of bacteria thriving in biofilms and working in concert with immunological susceptibilities in the host. In particular, periodontal diseases are a group of infections that lead to damage of the periodontium and ultimately, exfoliation of the teeth, and these infections are among the most common bacterial diseases of humans [8,9]. As complex as these multifactorial inflammatory diseases are, there is a consistent relationship between the Gram-negative anaerobe *Porphyromonas gingivalis *and severe, chronic manifestations of the disease [8-11]. However, while bacteria have traditionally been viewed as strictly beneficial or harmful, it is our contention that these over-simplified designations are no longer appropriate, and that expression of an organism's potential pathogenicity is context dependent [12]. The current genomic revolution offers an unprecedented opportunity to identify the molecular foundations of host-commensal and host-pathogen relationships so that we can understand how they assist or interfere with our normal physiology [2]. In line with a broader contextual view of relative potential pathogenicity, transcriptional profiling has emerged as a tool that allows the host to report the level of disruption induced by bacteria in the absence of preconceived notions regarding bacterial "intentions" (reviewed in [12,13]).

The initial interface between periodontopathic organisms, such as *P. gingivalis*, or members of the normal flora such a *Streptococcus gordonii *and the host is the epithelial layer in the subgingival crevice. Epithelial cells have traditionally been considered a passive barrier to infection. There is, however, growing evidence that they contribute more significantly to host defense than previously appreciated. There is a significant body of literature that reports that these cells efficiently signal a microbial intrusion to the immune cells to ensure effective mobilization of the innate and specific defense mechanisms. Furthermore epithelial cells can produce oxidants and antimicrobial peptides to actively participate in fending off intruding microbes [14,15]. Consequently, several organisms have evolved to circumvent the specific defenses available to the epithelium. For example, *P. gingivalis *invades epithelial cells and remains viable intracellularly in a rather covert manner whereby host cell programmed cell death is suppressed [16]. *P. gingivalis *stimulates integrin-dependent signaling in host cells to effect invasion and subsequently resides in the perinuclear area in epithelial cells [17]. In fact, besides *P. gingivalis*, an intracellular location has been suggested to be a natural component of the lifestyle of a number of other oral organisms [18-20]. Consequently, the regulation of normal processes such as cell division or apoptosis may be key events to a balanced longstanding intracellular state whereby microbes of the oral cavity and host cells co-exist and inflict a minimal degree of harm on each other.

In regards to the extracellular source for this pathogen, numerous studies demonstrate that *P. gingivalis *aggregates with common members of supragingival plaque including the periodontal pathogen *Aggregatibacter actinomycetemcomitans*, commonly associated with Localized Aggressive Periodontitis (LAP), and numerous oral streptococci ubiquitous to early plaque biofilms [21-23]. Interestingly, *P. gingivalis *only forms biofilms with *S. gordonii *and other oral streptococci but not *S. mutans *species [24,25], the specific molecular adherence strategies of which are well documented [24,26-29]. Recently, it was discovered that approximately 1% to 2% of the *P. gingivalis *genome was regulated during the initial stages of development of a community with *S. gordonii *and contribute to the development of heterotypic biofilms [26]. Pioneering organisms and their interspecies interactions thus ultimately determine the diversity potential of heterotrophic biofilms developing on newly acquired tooth pellicle. One cannot consider the pathogenicity of single periodontal organisms without considering the biofilm context that brought them to the host interface. The encounter between host and microbiota may thus be a finely tuned set of interactions whereby both cell types co-exist with each other. Hence, the transcriptional status of gingival cells has significance for the specific response to individual organisms and to overall well-being of the host (reviewed in [12,13]).

This is the first report that determines the epithelial transcriptional response to co-culture with complex microbiota constituted with *P. gingivalis *and *S. gordonii*, two interacting co-inhabitants of the oral cavity. Colonization of the oral cavity with *S. gordonii *and related streptococci is one of the first steps in the development of plaque upon the acquired pellicle of a recently cleaned tooth structure. *P. gingivalis*, a later colonizing oral inhabitant, is associated with chronic gingival disease and can result in severe periodontal tissue destruction. The host response to oral colonization has both pathophysiological and temporal effects on disease progression induced by late colonizing pathogenic species. It is often the balance between commensal, opportunistic, and pathogenic species in a particular microbiota that can tip the scales in favor of periodontal health or disease [30]. Studying the patterns of gene expression induced by co-infection with a complex microbiota can help reflect a more biologically and physiologically relevant host-pathogen interplay. We investigated whether the presence of an oral commensal in co-culture with gingival epithelial cells and *P. gingivalis *in a mixed microbial challenge would impact the epithelial response. The data presented herein supported the concept that *S. gordonii *does impart profound and global transcriptional changes in the epithelial response to a *P. gingivalis *challenge. In addition, colonization with commensal species directly modulated the host cell's response to co-infection with a pathogenic species and antagonized the ability of *P. gingivalis *to modulate cell cycle, yet had little effect of the invasiveness of this pathogenic species.

## Results

### Impact of Co-Culture on Adhesion and Invasion Levels of Bacteria to HIGK

Many studies have shown that *S. gordonii *and *P. gingivalis *demonstrate vastly different interaction characteristics with gingival epithelial cells. The former aggregates on the outside of epithelial cells and the latter invades within twenty minutes of infection, and resides in a perinuclear localization [31-33]. The interaction characteristics of *P. gingivalis *with HIGK in mono- and mixed cultures were first investigated to establish the baseline of this system. Consistent with previous reports, at a Multiplicity of Infection (MOI) of 2500:1 and in mono-infection, a great majority of the *S. gordonii *remained extracellular and interacted to a total level of 20 ± 1 colony forming units (CFU) per HIGK cell. In contrast, *P. gingivalis *was recovered mostly intracellularly under the conditions used in this study, and at a level of approximately 25 ± 1 CFU per HIGK cell. To determine whether the level of invasion by *P. gingivalis *was impacted by co-culture in the presence of commensal species, antibiotic protection assays were conducted on cells co-infected with *P. gingivalis *and *S. gordonii *using the same infection parameters as in the mono-species infection experiments. Although invasion levels of *P. gingivalis *were reduced by co-infection with *S. gordonii*, the effect was not statistically significant. Similarly, the levels of adhesion of *S. gordonii *were not significantly impacted by co-culture with *P. gingivalis *(data not shown). Under the experimental conditions used herein, the HIGK monolayer remained of a normal appearance and morphology and did not appear to be affected by the co-culture with either of the bacterial species at 2 h post-infection. Microscopic observations supported the finding that co-culture with a complex flora, neither abrogated nor potentiated the invasiveness of *P. gingivalis *(data not shown). Hence, bacteria-bacteria interactions that occur in this complex flora model and under the conditions used herein did not significantly impact the invasive phenotype of *P. gingivalis*.

### A Complex Infection Elicits a Different Epithelial Transcriptome

**Figure 1:**
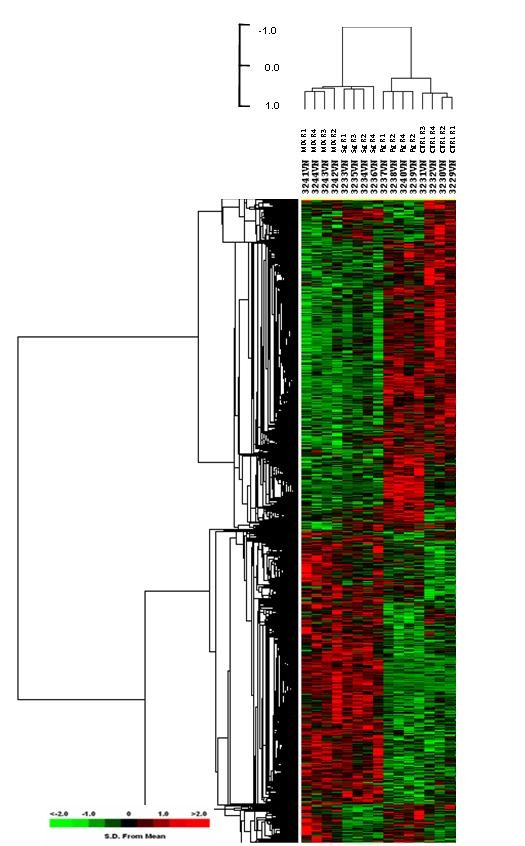
**HIGK Gene expression upon *P. gingivalis *and *S. gordonii *single- and mixed infections**. Hierarchical clustering was performed on variance-normalized signal of gene expression data from uninfected HIGK cells (CTRL) and from cells in co-culture with either organism or a mixture of both for 2 h before RNA isolation and purification. Probe set signal intensities were variance-normalized, mean-centered across samples, and subjected to hierarchical cluster analysis. Heat map and dendrogram were constructed from 6066 probe sets that were differentially expressed among the treatment conditions at a level of significance of *p *< 0.05. The degree of similarity between the transcriptional profiles of each sample is expressed by Pearson's correlation coefficient distance metric, according to the adjacent scale. The expression state of each data point is represented as standard deviations from the mean expression level for that gene in all samples. Red indicates a relative increase, green indicates a relative decrease, and black indicates no relative change of mRNA transcripts for a given gene.

Assessment of the HIGK transcriptional response during interaction with *P. gingivalis*, *S. gordonii*, or a mixture of the two organisms was performed to determine the global impact of a mixed microbial challenge on epithelial cells. Signal intensity data for the probe sets were categorized by unsupervised analysis using Cluster [34] and supervised class prediction with BRB Array Tools [35]. Relative intensity values were displayed using Treeview [34]. The unsupervised hierarchical clustering analysis showed that the biological replicates clustered together (data not shown). This demonstrated that each treatment condition generated a unique transcriptional response in HIGK that was consistent and reproducible amongst biological replicates. Supervised class prediction utilizing a combination of several prediction methods and a random variance model for univariate F tests [35] revealed 6066 probe sets were differentially expressed among the treatment conditions, at a level of significance of *p *< 0.05. Figure 1 shows a Treeview representation of the 6066 probe sets differentially expressed amongst the four treatments. A two hour mono-infection with *S. gordonii *and *P. gingivalis *alone demonstrated unique transcriptional responses that were diametrically opposite to one another, as well as distinct when compared to uninfected HIGK. At this early infection time point, the HIGK's transcriptional response to mixed infection appears most similar to the transcriptional pattern of the HIGK infected with *S. gordonii *alone. These data suggested that, under mixed infection conditions, the commensal species had the ability to inhibit and reprogram the transcriptional response elicited by *P. gingivalis*. The antagonistic effect of *S. gordonii *on the transcriptional response to cells co-infected with *P. gingivalis *could not be ascribed to different invasion levels of *P. gingivalis *in mixed challenges.

**Figure 2:**
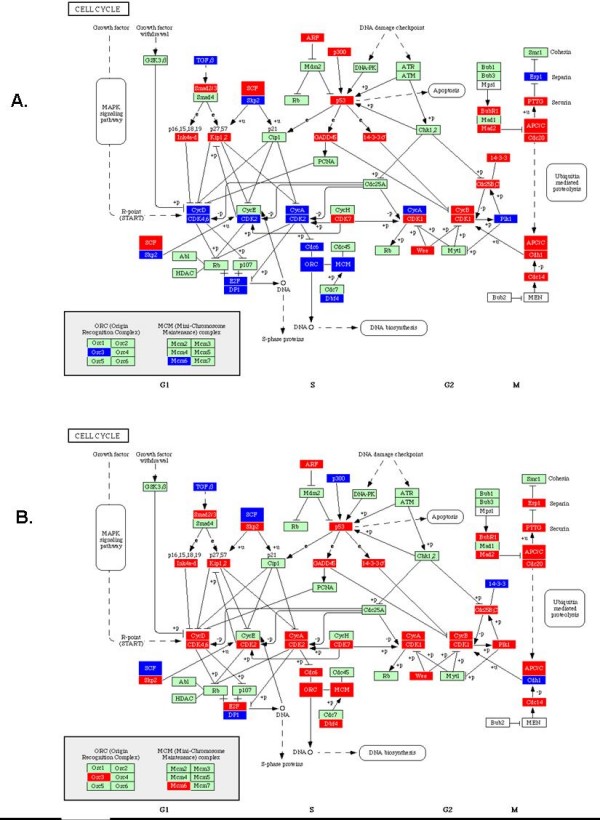
**Impact of a mixed microbial challenge on cell cycle control in HIGK**. Transcriptional regulation of the KEGG cell cycle pathway modulated by infection with *P. gingivalis *alone (A) or a mixture of *P. gingivalis *and *S. gordonii *(B). Genes represented by red boxes indicate up-regulated transcript levels compared to uninfected controls, where as those genes depicted in blue represent down-regulated transcript levels.

While mixed co-cultures of pathogen with commensal species clustered closer to the signatures associated with *S. gordonii *alone, ontology analysis was undertaken to elucidate the biological relevance of the cellular pathways most differentially impacted by a mixed infection condition. The 6066 probe sets that were differentially impacted at a significance level of *p *< 0.05 amongst the infection conditions were used to populate known KEGG pathways using Pathway Express software [36-39]. One feature of this software is the use of statistical algorithms to determine the most highly impacted pathways, considering the number of input genes compared to total genes in a given pathway; a commonly used hypergeometric over-representation approach. The most highly impacted pathways (*p *< 0.05) populated by genes differentially expressed (also *p *< 0.05) are listed in Table 1. As it was the most significantly impacted pathway in this analysis (*p *< 0.001), the cell-cycle pathway was selected for further phenotypic validation. As indicated in Table 1, a total of 45 cell cycle-associated genes were differentially modulated by a complex flora as compared to mono-infection with *P. gingivalis*. A significant proportion of two key classes of regulatory molecule genes – cyclins and their cognate cyclin-dependent kinases (CDK) – were differentially regulated in HIGK under each infection condition. Cyclins are transiently expressed nuclear proteins that are required for CDK activation that drives progression through a cell cycle [40]. A summary of the major cell cycle regulatory molecules impacted by infection with *P. gingivalis *and *S. gordonii *is presented in Figure 2.

**Table 1:** HIGK gene ontology comparing *P. gingivalis *and *S. gordonii *co-infectionto *P. gingivalis *mono-infection.^a^

**Impacted pathway**^**b**^	**Impact factor**^**c**^	**# of modulated genes (total) in Pathway**^**d**^	***p*-value**
Cell cycle	13.542	45 (112)	< 0.001
Ubiquitin mediated proteolysis	11.305	22 (45)	< 0.001
Adherens junction	8.788	29 (77)	< 0.001
TGF-beta signaling pathway	7.989	29 (84)	0.002
SNARE interactions in vesicular transport	7.691	16 (36)	0.001
Apoptosis	7.513	30 (84)	0.002
Jak-STAT signaling pathway	6.989	46 (153)	0.003
MAPK signaling pathway	6.207	75 (273)	0.009
Wnt signaling pathway	5.208	42 (147)	0.02
Colorectal cancer	5.184	23 (77)	0.05
Notch signaling pathway	5.134	16 (46)	0.03

^a^The epithelial cell pathways were determined by Pathway Express.

^b^Kyoto Encyclopedia of Genes and Genomes pathways .

^c^The impact factor measures the pathways most affected by the changes in gene expression by considering the proportion of differentially regulated genes, the perturbation factors of all the pathway genes, and the propagation of these perturbations throughout the pathway. Only pathways with an impact factor greater than 5 are included.

^d^Number of regulated genes in a pathway/total number of genes currently mapped to this pathway.

### Effect of a Mixed Flora Challenge on the Epithelial Cell Growth Dynamic

**Figure 3:**
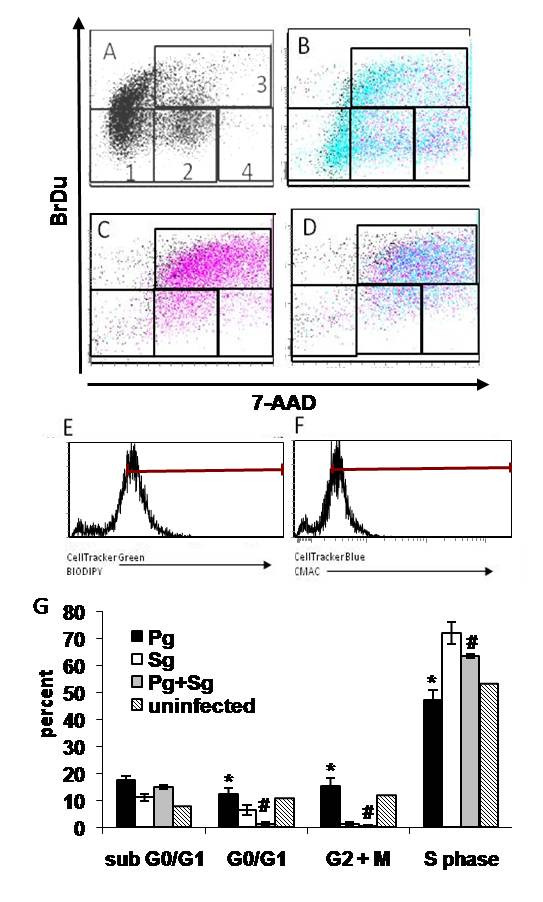
**Infection with *S. gordonii *antagonizes the effect of *P. gingivalis *on the cell cycle**. HIGK (uninfected in A) were simultaneously grown in the presence of BrdU and infected with *P. gingivalis *labeled with CellTracker Green BIODIPY shown in blue (B), CellTracker Blue CMAC labeled *S. gordonii *shown in pink (C), or a combination of *P. gingivalis *and *S. gordonii *for 4 h (D). HIGK cells were treated with antibiotic to kill extracellular bacteria and were cultured for an additional 20 h prior to harvesting for FACS analysis. The cell cycle positions and active DNA synthetic activities of cells were determined by analyzing the correlated expression of total DNA (7-AAD) and incorporated BrdU levels. Uninfected control HIGK were included for comparison of baseline cycling patterns. Data represent duplicate experiments. Region gate 1, HIGK were apoptotic (defined as sub G_0_/G_1_); region 2, G_0_/G_1_; region 3, S phase; region 4, G_2_+M. Panel E and F demonstrate the gated HIGK populations used throughout all the analyses for CellTracker Green labeled *P. gingivalis *and CellTracker Blue CMAC labeled *S. gordonii*, respectively. Panel G depicts quantitative analysis of HIGK cell cycling in response to mono- and mixed infections. Results are the mean of three experiments. Statistical analysis was conducted using an ANOVA with Bonferroni's Multiple Comparison Test. * Pg vs. Sg sub G_0_/G_1_, Pg vs. Sg G_0_/G_1_, Pg vs. Sg G_2_+M, Pg vs. Sg S phase, p value < 0.05. # Pg vs. Sg+Pg sub G_0_/G_1_, G_0_/G_1_, G_2_+M, S phase, p values < 0.001. Error bars represent the mean ± SD.

The finding that cell cycle regulation was significantly impacted in HIGK cells encountering mono- versus mixed infection was confirmed at the cellular level using two-color fluorescence-activated cell sorting (FACS) cytometric analysis. The percentage of HIGK in any given phase of the cell cycle was analyzed using HIGK co-cultured with *P. gingivalis *and *S. gordonii *in mono- and mixed-cultures in the presence of BrdU (Figure 3). The ratios of BrdU amounts incorporated during DNA replication of S phase, compared to total DNA content and overall cell size, reflects the cell cycle phase of a given cell. A minimum of 1 × 10^4 ^cells were analyzed in each condition to determine the proportion of HIGK in different cell cycle phases (Figure 3, panel G). The greatest differences infection status imparted upon HIGK cell cycling were observed during S phase, which was consistent with the mRNA profiling where high proportion of S-phase promoting factor genes (Figure 3) were impacted by infection with a mixture of *S. gordonii *and *P. gingivalis*, compared to mono-infection with pathogen alone. Infection with *S. gordonii *resulted in the majority of HIGK remaining in S phase while infection with *P. gingivalis *resulted in a wider distribution of HIGK in each of the various stages of cell cycling, mirroring uninfected controls. Introduction of the commensal *S. gordonii *to *P. gingivalis*-infected HIGK cells promoted progression of HIGK into S-phase of the cell-cycle, shifting the cycling trend towards that of cells challenged only with the commensal species (Figure 3). What remained consistent amongst the microarray and phenotypic experiments was that HIGK infected with a mixture of *S. gordonii *and *P. gingivalis *cycled similarly to cells mono-infected with *S. gordonii*.

**Figure 4:**
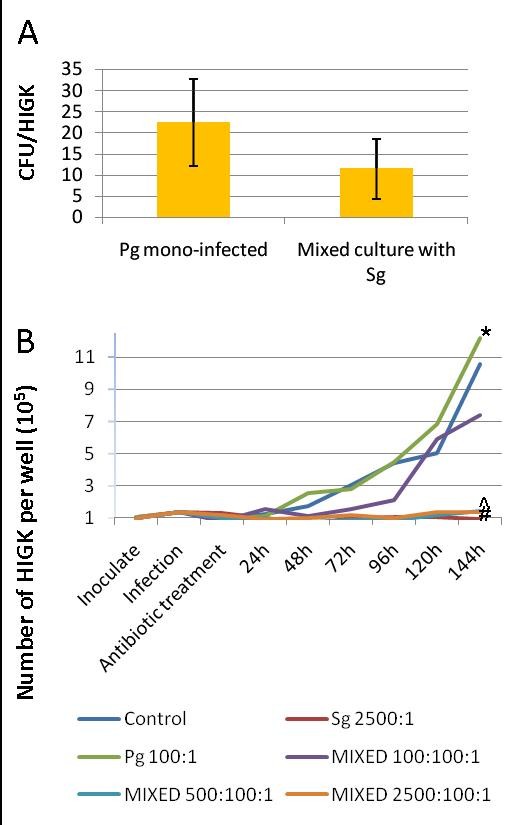
**Accelerated HIGK proliferation upon infection with *P. gingivalis *is inhibited in co-culture with *S. gordonii***. **A) **Total interaction and invasion levels determined by measuring the total numbers of *P. gingivalis *associated with HIGK cells by live counts in mono-(Pg) and in mixed infection (Mixed) with *S. gordonii *at the highest MOI. Results are the mean of three experiments. **B) **HIGK cells at a low confluency were co-cultured with single and complex mixtures of bacteria and cultured for up to 144 hours. Labels: Control, uninfected HIGK; Sg, *S. gordonii*; Pg, *P. gingivalis*; MIXED, co-infection with *S. gordonii *and *P. gingivalis *at different multiplicity of infection (MOI). All cell counts were performed in triplicate and all experiments were repeated twice. * *p *> 0.05 *P. gingivalis *vs non-infected controls; #*p *< 0.05 *S. gordonii *vs. non-infected controls; ^*p *< 0.05 *S. gordonii *co-infection with *P. gingivalis *(2500:1) vs. non-infected controls (*p *< 0.05) by ANOVA with Dunnett's Multiple Comparison Test.

FACS analysis confirmed microscopic observations and CFU data on *P. gingivalis *interaction characteristics with HIGK. As shown in Figure 3 (blue dots), the level of total interaction was similar (*p *= 0.106) whether *P. gingivalis *was in single (B) or in mixed (D) co-cultures with HIGK. Thus, both the total numbers of *Pg *interacting per cell (Figure 4A), as well as the total numbers of HIGK that interact with at least a single *Pg *bacterium (Figure 3B, D) were both not significantly influenced by *Sg *coinfection compared to mono- *Pg *infection. This corroborates the notion that the observed shift in cycling behavior of HIGK co-cultured with *P. gingivalis *and *S. gordonii *compared to that of HIGK infected with *P. gingivalis *alone was not due to the inhibition of *P. gingivalis *adhesion or invasion. Transcriptionally at 2-hours, and phenotypically at 24 h post-infection, commensal species in a mixed infection can thus influence HIGK's physiological response towards one that is characteristic of HIGK cells mono-infected with a commensal species.

To reconcile the transcriptional snap-shot and the cell-cycle analysis into a coherent model, a direct measurement of cell division time was performed over a period of six days after a 2 h infection. As shown in Figure 4B, mono-infection with *P. gingivalis *stimulated an HIGK growth rate resembling that of uninfected controls (*p *> 0.05). In contrast, mono-infection with *S. gordonii *constrained growth of HIGK as compared to non-infected controls (*p *< 0.05). Mixed infection of HIGK with *S. gordonii *and *P. gingivalis *resulted in a dose-dependant inhibition of cell growth where *S. gordonii *antagonized the growth stimulatory effect of *P. gingivalis *at the lowest MOI tested (100:1), but seen with more significance at higher MOIs (500:1) (2500:1) (*p *< 0.05). The constrained growth observed under *S. gordonii *infection conditions was not due to indirect effects, such as pH changes or nutrient depletion of the growth media, but are the result of direct interaction between *S. gordonii *and HIGK cells because conditioned *S. gordonii *media had no effect on HIGK proliferation (see Additional file 1, Additional file 2, and Additional file 3).

## Discussion

Previous studies have elucidated the unique transcriptional signatures that are elicited in response to mono-infection with oral pathogens *P. gingivalis *and *A. actinomycetemcomitans *[41] as well as opportunistic bacteria such as *Fusobacterium nucleatum *and the commensal species including *S. gordonii *[42]. Transcriptional responses to *P. gingivalis *or *A. actinomycetemcomitans *mono-infections demonstrated organism-specific responses that are drastically different [41]. In contrast, the opportunistic pathogen *Fusobacterium nucleatum *and *S. gordonii *elicit profiles that are more transcriptionally restrained in comparison to those characterizing the overt periopathogens [42]. Interestingly, the transcriptional changes that have been observed in HIGK infected with different commensal or pathogenic species does not correlate with the levels of invasiveness that are characteristic of these bacterial species. For example, *F. nucleatum *invades epithelial cells, while *S. gordonii *does not. Yet, both species induce very similar transcriptional responses in HIGK [42]. This considerable overlap suggests an evolutionary-driven programmed response to the presence of indigenous constituents of the normal human oral flora. Hence, gingival epithelial cells can differentiate between commensal or potentially pathogenic species, regardless of their cellular localization, and respond accordingly. In this study, the dichotomy in the responses to pathogen or commensal supports previous reports that suggested a species-specific recognition that was tailored to each different bacterial species [41,42].

The microbial challenge faced by the subgingival area is one of great complexity and is dynamic in status. The host epithelial response to *in vivo *oral biofilms would be extremely difficult to mimic, as an *in vitro *biofilm model may not exactly duplicate the complex microbiome present in the oral cavity. Furthermore, the strata of organisms present in oral biofilms/plaque vary widely amongst individuals [43]. As such, there are few studies that have investigated bacterial pathogenicity in complex infection models. Nevertheless, the current work offers proof-of-principle that an admittedly overly simplified mixed microflora can have profound effects on the host transcriptome, as compared to mono-species bacterial challenges. To date, this is the first characterization of the epithelial response to a mixed infection encompassing commensal and pathogenic organisms. When *S. gordonii *and *P. gingivalis *were co-cultured together with HIGK, the resulting transcriptional response of the host cell was most similar to that elicited by infection with the *S. gordonii *alone. This finding supports the hypothesis that commensal species modulate the pathogenicity of *P. gingivalis in vivo*. This result was not surprising considering that microbe-microbe interactions between *P. gingivalis *and *S. gordonii *modulate the gene expression pattern of *P. gingivalis *[26].

In our model, the most significant effect of a mixed challenge was the impact on host cell cycling. HIGK infected with *P. gingivalis *were more evenly distributed in all phases of the cell cycle compared to cells infected with both *P. gingivalis *and *S. gordonii*. HIGK exposed to this mixed co-culture infection, as well as *S. gordonii *alone were more prevalently found in Synthesis phase. Whether this is a true stage arrest phenomenon elicited by the presence of *S. gordonii *could not be determined since cells were not *a priori *synchronized. Nevertheless, cell growth analysis corroborated the ability of *P. gingivalis *to induce cellular proliferation as compared to uninfected controls. Further, mixed infections with *S. gordonii *were capable of inhibiting the cellular proliferation induced by *P. gingivalis *in a dose-dependant fashion.

Our microarray analyses are in line with previous proteomic experiments performed in primary gingival epithelial cells (GEC) [44]. Both studies found that several pathways exerting regulatory control over the cell cycle were impacted by *P. gingivalis *mono-infection. Both studies also consistently showed a regulation of Cdk2 and Cdk4/6 upon infection of epithelial cells with *P. gingivalis*. Both Cdk2 and Cyclin D were down-regulated in response to infection with *P. gingivalis *with respect to transcript levels and protein levels. Conversely, transcript levels were increased in response to a mixed infection with *S. gordonii*. Only by combining genomic, proteomic, and phenotypic epithelial responses can a true picture emerge of what impact the increasing complexity of a microbiome has on host cellular responses.

Recent evidence supports the concept that cell cycle is significantly impacted in diseased sites as compared to matching healthy sites in periodontitis and gingivitis patients [45]. Although arguably more clinically relevant, studies involving human specimens or primary cell culture present the greatest potential for uncontrolled experimental variables. Examples include the genetic variability between donors, different levels of inflammation and age of participant, diet, diurnal variations in gene expression, type of anesthesia used, length of ischemia prior to tissue removal, time from tissue removal to RNA stabilization, and other confounding factors [12]. In contrast, experiments performed with immortalized cell cultures – although simplistic models of the *in vivo *environment – present a significantly higher degree of stability and can be manipulated. This translates into a dataset that is less noisy and ultimately presents a greater potential to dissect a given pathway and predict a phenotype with biological relevance. Hence, the current transcriptional dataset provided valuable insight on the intricate and complex mechanisms that may be responsible for the differential cell cycle effect of *P. gingivalis *and *S. gordonii*, and provided clues on how the presence of the latter in a mixed infection may affect the proliferative properties of *P. gingivalis*. For example, CycD, CDK4 and CDK6, were upregulated in HIGK infected with the mixed microflora compared to HIGK transcript levels in response to mono-infection with *P. gingivalis*. At the beginning of the cell cycle, Cyclin D and CDK4 and 6 form complexes in response to extracellular signals for growth and stimulate entry into G_1_. The same pattern held for CDK2 which complexes with several different cyclins, but when bound to cyclin E pushes the cell from G_1 _to S phase (G_1_/S transition). Cyclin A, which when complexed with CDK2 initiates the G_2_/M transition, was upregulated in the presence of a mixed infection of HIGK. Transcript levels of all of these cell cycle regulators were down regulated in *P. gingivalis *infected cells compared to uninfected controls. Down regulation of Cyclin A transcript in *P. gingivalis *infected HIGK fits with regulation of the rest of the cell cycle modulating factors because Cyclin A, when complexed with CDK1, also functions as a S-phase promoting factor. Many regulatory steps in protein production, folding, and function as well as post-transcriptional kinase activities can also impact protein function. These post-transcriptional modifications were not detected by transcriptional profiling, yet may significantly alter the initial epithelial transcriptional response with any bacterium. While the aforementioned cyclins and CDKs play a large role in determining the stage of cell cycle progression, ultimately these regulatory molecules control the activity of the transcriptional activator E2F. The activation of E2F results in transcription of a number of genes that promote the cell's transition from G_1 _to S phase via phosphorylation and deactivation of the Rb protein. E2F transcript levels were up-regulated in HIGK infected with both *P. gingivalis *and *S. gordonii *and down-regulated in cells mono-infected with *P. gingivalis*. CDC6, which is required for the initiation of DNA replication, was similarly regulated in both treatment conditions. Further validation of the current transcriptional dataset is ongoing in primary GECs utilizing clinical biofilm specimens.

Besides the cell cycle pathway, additional pathways were differentially impacted by mono- versus mixed infections, and are currently being further confirmed at the protein level and phenotypically. These pathways include apoptosis and numerous signaling pathways (Table 1). In addition, genes associated with cancer were differentially impacted by infection with *P. gingivalis*, which is not surprising since genes involved in normal physiological functions are often also implicated in cancer when they are disregulated. It remains unclear and speculative whether *P. gingivalis *is directly involved in the initiation or exacerbation of carcinogenic lesions or whether this effect is consequent to the attempt of *P. gingivalis *to establish an anti-apoptotic phenotype in GECs. This phenomena has already been shown to help this microorganism to propagate a suitable niche for an extended infection and involves manipulating pathways that are normally involved in normal cellular functions as well as in cancer [16,41,42,46,47]. Hence, further endeavors into the possible carcinogenic potential of periodontal pathogens and the dissection of the role of commensal species in affecting these pathways is appropriate and timely.

## Conclusion

In summary, the present study provides evidence that the degree of complexity of a mixed microbiota influences the transcriptional response to infection of host epithelial cells. The transcriptional repertoire of genes impacted by co-infection with *S. gordonii *and *P. gingivalis *compared to that of HIGK infected with *P. gingivalis *alone demonstrated that commensal species are able to modulate expression of host genes with a broad diversity of physiological functions and antagonize the effect of pathogenic species at the cellular level. This global expression study provides insight into both host-pathogen interactions within the context of a complex microbial infection and challenges the current dogma regarding the *potential *versus the *actual *pathogenicity of an oral species in the context of a complex biofilm. Although *S. gordonii *is consistently referred to as a commensal, its association with the host is not completely inconsequential as it serves as an early colonizer responsible for the development of potentially pathogenic plaque species. The study of the global transcriptome of epithelial cells to single and complex microbial challenges offers clues towards a better understanding of how bacteria-bacteria interactions and bacteria-host interactions impact the overall host response. Ultimately, this work could lead to identifying host responses that can be used to predict a commensal or pathogenic challenge as well as the overall pathogenic potential of a mixed microbiota. Genes and cell pathways impacted by such infections could serve as diagnostic markers to assess the pathogenicity of individual complex microbial communities across various populations battling different manifestations and stages of periodontal disease.

## Methods

### Bacterial strains

*Porphyromonas gingivalis *strain ATCC 33277 was cultured anaerobically for 24 h at 37°C in trypticase soy broth supplemented with yeast extract (1 mg mL^-1^), haemin (5 μg mL^-1^), and menadiaone (1 μg mL^-1^). *Streptococcus gordonii *strain DL1 was cultured aerobically for 24 h at 37°C in Todd Hewlett Broth supplemented with 0.5% yeast extract and 0.5% glucose.

### Eukaryotic cell lines

Human immortalized gingival keratinocytes (HIGK) were originally generated by transfection of primary GEC with E6/E7 from HPV [48]. HIGK were cultured under 5% CO_2 _in keratinocyte serum-free medium (K-SFM, Gibco/Invitrogen, Carlsbad, CA) supplemented with 0.05 mM calcium chloride, 200 mM L-glutamine (Gibco/Invitrogen), and 1% antibiotic/antimycotic (Gibco/Invitrogen).

### Microbial-host cell co-culture

Bacteria were harvested by centrifugation and resuspended in antibiotic-free K-SFM media. HIGK (10^7 ^cells) were washed three times with phosphate-buffered saline (PBS) and incubated with bacteria at an MOI of 100 for *P. gingivalis *and 2500 for *S. gordonii*. After 2 h at 37°C, the innoculate was removed and HIGK cells were immediately lysed with Trizol reagent (Invitrogen, Carlsbad, CA) before RNA extraction. Co-cultures were carried out in quadruplicate per infection condition. For microbial-host cell interaction studies, total numbers of *S. gordonii *associated with HIGK – both externally and internally – after 2 h incubation and washing were determined by Triton X-100 lysis and plate counts. Invasion levels of *S. gordonii *and *P. gingivalis *co-cultured with HIGK both individually and together in a mixed infection were measured by antibiotic protection assays as previously described [49]. In mixed co-culture with HIGK and *P. gingivalis *mono-infection, cells were lysed with sterile water as *P. gingivalis *were not viable after exposure to Triton X-100.

### RNA isolation, cRNA synthesis and chip hybridization

Total RNA was extracted, DNAse-treated, purified and quantified according to standard methods (Qiagen and Affymetrix). Briefly, double stranded cDNA was synthesized using 8 μg of total cellular template RNA according to standardized protocol (SuperScript double stranded cDNA synthesis kit; Invitrogen, Carlsbad, CA). cRNA was transcribed *in vitro*, incorporating biotinylated nucleotides via a BioArray high-yield RNA transcript labeling kit (T7) (Enzo Life Sciences, Farmingdale, NY), fragmented, and hybridized onto the Affymetrix human genome U133 Plus 2.0 human microarrays. Each condition was studied in biological quadruplicate and samples were not pooled. The microarrays were hybridized for 16 h at 45°C, stained with phycoerythrin-conjugated streptavidin and washed according to the Affymetrix protocol (EukGE-WS2v4) using an Affymetrix fluidics station, and scanned with an Affymetrix scanner.

### Microarray data analysis

Microarray data analysis was performed as previously described [41,42]. Briefly, expression filters were applied to remove Affymetrix controls and probe-sets whose signal was undetected across all samples. The signal intensity values of the resulting dataset were variance-normalized, mean-centered and ranked by their coefficients of variation. Normalization was performed to give equal weight to all probe-sets in the analysis, regardless of the order of magnitude of the raw signal intensity. To reduce the confounding effect of background signal variation on the analysis, only the half of the dataset demonstrating the most variation across samples was used to perform unsupervised hierarchical cluster analysis using Cluster software [34]. The resulting heat-map and cluster dendrograms were visualized with Treeview software [34] to reveal the extent of characteristic host cell responses to each infection state, defined as identical treatments clustering together. Array results have been deposited in the GEO repository  under accession number GSE12121. Following initial assessment of the host cell response to each condition, supervised class prediction was performed to investigate differences in gene regulation among experimental conditions. For this analysis, the raw signal intensities were log-transformed for all probe-sets that passed the initial expression filters, and were correlated using BRB Array Tools [35]. Diagonal linear discriminant analysis, 1- and 3- nearest neighbors, and nearest centroid prediction methods were used in conjunction with a random variance model for univariate F tests. In each supervised analysis, biological replicates were grouped into classes according to their infection state during co-culture experiments and probe sets significant at the *p *< 0.05 level between the class were identified. To test the ability of these significant probe sets to truly distinguish between the classes, leave-one-out-cross-validation (LOOCV) studies were performed for each prediction model. In these LOOCV studies each array was left out in turn and classifiers were derived from the remaining 15 samples. The ability of these classifiers to correctly predict the identity of the left out sample was compared to the expected rate due to chance alone (25% for 4 classes). Using the gene expression classifiers derived from linear discriminant analysis and 1-nearest neighbor methods, the arrays were correctly classified 75% of the time. Classifiers from 3-nearest neighbors and nearest centroid correctly predicted the identity of the left-out array with 69% accuracy. To assess the significance of the LOOCV results, Monte Carlo simulation with 2,000 random permutations of the dataset was also performed. The significance of the LOOCV results was *p *< 0.001 for all prediction methods. Characterized KEGG pathways were populated by the resulting list of probesets significant at the *p *< 0.05 level (6066) using Pathway Express, available at [36-39]. The *p *values for each pathway were calculated using hypergeometric over representation approach with Bonferroni's Multiple Comparisons Test.

### Cell cycling analysis of infected HIGK

*P. gingivalis *were labeled overnight using live dye CellTracker Green BODIPY (Molecular Probes). *S. gordonii *were labeled with live dye CellTracker Blue CMAC (Invitrogen) in a similar manner. HIGK were infected with the same MOI used in the microarray experiments in the presence of 1 mM BrdU for four hours prior to treatment with antibiotic/antimycotic and gentamicin (300 μg mL^-1^) to kill extracellular bacteria. Cells were then allowed to grow an additional twenty hours before harvesting for FACS analysis. For quantitative analysis, similar infection conditions were used but only *P. gingivalis *was labeled with CellTracker Green BODIPY.

APC BrdU Flow kits were purchased from BD Pharmingen (San Diego, CA) and used for flow cytometric analysis of cell cycle. Briefly, bromodeoxyuridine (BrdU)-pulsed cells were fixed and permeabilized with BD cytofix/cytoperm buffers, after which DNAse was used to expose incorporated BrdU. APC conjugated anti-BrdU was used to label newly incorporated BrdU while 7-amino-actinomycin D (7-AAD) was used to stain total DNA content. Data was acquired using a FACS Calibur (Becton Dickinson, Mountain View, CA) and analyzed using FCS express software (De Novo). Three independent experiments were performed of each condition in triplicate. Statistical analyses were performed using an ANOVA with Bonferroni's Multiple Comparison test.

### HIGK growth analysis

Approximately 10^5 ^HIGK (ca. 10% confluence) were seeded to T75 flasks in K-SFM with supplements. The cells were co-cultured with single and complex mixtures of bacteria at 37°C in 5% CO_2 _for 2 h at various MOI and under the conditions described above. After infection, the cells were washed three times with PBS and further cultured for up to 144 hours in K-SFM supplemented with antibiotic/antimycotic (Gibco/Invitrogen) and gentamicin (300 μg mL^-1^). At each time-point, the cells were dissociated using Accutase (Innovative Cell Technologies, San Diego, CA) following the manufacturer's recommendations and cell counts were determined using a Z1 Coulter Particle Counter (Beckman/Coulter). All cell counts were performed in triplicate and all experiments were repeated twice. ANOVA with Dunnett's Multiple Comparison Test was used to determine statistical significance of each infection condition compared to uninfected controls.

## List of Abbreviations

(7-AAD): 7-amino-actinomycin D; (BrdU): bromodeoxyuridine, (CFU): colony forming units; (cDNA): complementary DNA; (cRNA): complementary RNA; (CDK): cyclin-dependent kinase; (DNA): deoxyribonucleic acid; (FACS): fluorescence-activated cell sorting; (G_0_, G_1_, G_2_): gap/growth phase; (GEC): gingival epithelial cells; (HIGK): human immortalized gingival keratinocytes; (HPV): human papillomavirus; (IL-8): interleukin 8; (K-SFM): keratinocyte serum free media; (LOOCV): leave-one-out cross-validation; (LAP): Localized Aggressive Periodontitis; (M): mitosis; (MOI): multiplicity of infection; (PBS): phosphate-buffered saline; (RNA): ribonucleic acid; (S): synthesis phase.

## Authors' contributions

JJM and KvL contributed equally to this work and should be considered co-first authors. JJM participated in the design of the study, aided the microbial-host cell co-culture studies, aided with all microarray experiments and analysis, performed the supplemental HIGK growth analyses experiments, and helped draft the manuscript. KvL participated in the design of the study, carried out the microbial-host cell co-culture studies, the cell cycling analysis and drafted the manuscript. CD conducted confocal laser fluorescence microscopy experiments (data not shown). SW aided in cell cycling analysis. SMW participated in design of the cell cycling analyses, performed the statistical analysis, and helped to draft the manuscript. HVB helped coordinate the microarray data analysis. RJL participated in the design of the study and helped to draft the manuscript. MH conceived the study, participated in its design and coordination, performed the microarray statistical analysis, conducted the HIGK growth analyses experiments, and helped to draft the manuscript. All authors read and approved the final manuscript.

## Supplementary Material

Additional file 1 **"Antagonism of *P. gingivalis*-induced HIGK proliferation by *S. gordonii *is not due to indirect effects upon culture media." **line graph showing HIGK cell growth over time under 6 experimental conditions. Click here for file

Additional file 2 **"Antagonism of *P. gingivalis*-induced HIGK proliferation by *S. gordonii *is not due to indirect effects upon culture media." **word document describing the experimental conditions used to generate file 1. Click here for file

Additional file 3 **"Supplementary Materials and Methods. Antagonism of *P. gingivalis*-induced HIGK proliferation by *S. gordonii *is not due to indirect effects upon culture media." **word document describing the materials and methods used to generate file 1/supplementary figure. Click here for file
