# Supplementary figures and images for: The degree of microbiome complexity influences the epithelial response to infection
Source: BMC Genomics. 2009 Aug 18;10:380. doi: 10.1186/1471-2164-10-380 (PMC2736203; doi:10.1186/1471-2164-10-380)

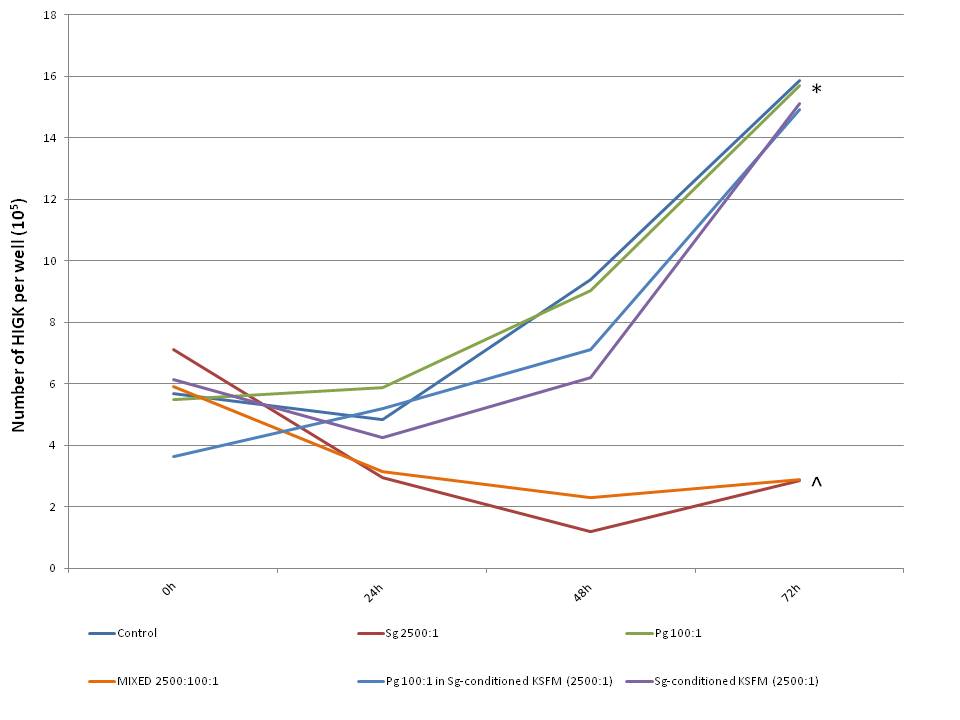

Supplement: Additional file 1 — "Antagonism of P. gingivalis-induced HIGK proliferation by S. gordonii is not due to indirect effects upon culture media." line graph showing HIGK cell growth over time under 6 experimental conditions. [file 1471-2164-10-380-S1.jpeg]
