# Supplementary material for: The degree of microbiome complexity influences the epithelial response to infection
Source: BMC Genomics. 2009 Aug 18;10:380. doi: 10.1186/1471-2164-10-380 (PMC2736203; doi:10.1186/1471-2164-10-380)
Supplement: Additional file 2 — "Antagonism of P. gingivalis-induced HIGK proliferation by S. gordonii is not due to indirect effects upon culture media." word document describing the experimental conditions used to generate file 1. [file 1471-2164-10-380-S2.doc]

**Supplementary Figure – Antagonism of *P. gingivalis*-induced HIGK proliferation by *S. gordonii* is not due to indirect effects upon culture media.**

HIGK cells at a low confluency were co-cultured with various combinations of *S. gordonii* (*Sg*), *P. gingivalis* (*Pg*), and *Sg*-conditioned media to assess HIGK response to single or mixed infection and cultured for up to 72 hours. Labels: **Control**, uninfected HIGK; **Sg 2500:1**, infection with *S. gordonii* at multiplicity of infection (MOI) 2500:1; **Pg 100:1**, infection with *P. gingivalis* MOI 100:1; **MIXED 2500:100:1**, simultaneous co-infection with *S. gordonii* and *P. gingivalis* at MOI 2500*Sg*:100*Pg*:1; **Pg100:1 in Sg-conditioned KSFM (2500:1)**, infection with *P. gingivalis* at MOI 100:1 in media conditioned by *Sg* growth for 2 hours using an equivalent number of *Sg* to attain 2500:1 MOI if bacteria were not removed; **Sg-conditioned KSFM (2500:1) )**, infection with media conditioned by *Sg* growth for 2 hours using an equivalent number of *Sg* to attain 2500:1 MOI if bacteria were not removed. 2. All cell counts were performed in triplicate and all experiments were repeated twice. * *p*>0.05 Pg100:1 vs. non-infected controls, Pg100:1 in Sg-conditioned KSFM (2500:1) vs. non-infected controls, Sg-conditioned KSFM (2500:1) vs. non-infected controls; ^ *p*<0.001 *S. gordonii* (2500:1) vs. non-infected controls; *S. gordonii* (2500:1) co-infection with *P. gingivalis* (100:1) vs. non-infected controls. (*p*<0.001) by ANOVA with Dunnett’s Multiple Comparison Test.
