# Supplementary material for: The degree of microbiome complexity influences the epithelial response to infection
Source: BMC Genomics. 2009 Aug 18;10:380. doi: 10.1186/1471-2164-10-380 (PMC2736203; doi:10.1186/1471-2164-10-380)
Supplement: Additional file 3 — "Supplementary Materials and Methods. Antagonism of P. gingivalis-induced HIGK proliferation by S. gordonii is not due to indirect effects upon culture media." word document describing the materials and methods used to generate file 1/supplementary figure. [file 1471-2164-10-380-S3.doc]

**Supplementary Materials and Methods**

**Antagonism of *P. gingivalis*-induced HIGK proliferation by *S. gordonii* is not due to indirect effects upon culture media.**

To isolate any potential indirect effects speculated to exist, KSFM was conditioned by *S gordonii* under conditions identical to a co-culture experiment and in the absence of HIGK cells. After 2 hours incubation of the bacteria in KSFM (37^o^ C and 5% CO_2_), the media was collected and the bacteria were removed by two consecutive centrifugation steps of 3000 rcf for 10 minutes, where the supernatant was transferred to a clean tube and bacterial pellet discarded. This conditioned media was then used to repeat the HIGK proliferation experiments which quantified the result of *S. gordonii*-induced cell cycle arrest. The added conditions of “*S. gordonii*-conditioned media” and “*P. gingivalis* resuspended in *S. gordonii*-conditioned media” were used in conjunction with “non-infected controls,” “*Sg* single infection (2500:1 MOI),” “*Pg* single infection (100:1 MOI),” and “Mixed infection (*Sg* 2500:*Pg* 100:1 MOI).”

Approximately 10^5^ HIGK (ca. 10% confluence) were seeded to T75 flasks in K-SFM with supplements. The cells were co-cultured with single and complex mixtures of bacteria or conditioned media at 37°C in 5% CO_2_ for 2 h under the conditions described above. After infection, the cells were washed three times with PBS and further cultured for up to 72 hours in K-SFM supplemented with antibiotic/antimycotic (Gibco/Invitrogen) and gentamicin (300 μg mL^-1^). At each time-point (0h, 24h, 48h, 72h), the cells were dissociated using Accutase (Innovative Cell Technologies, San Diego, CA) following the manufacturer’s recommendations and cell counts were determined using a Z1 Coulter Particle Counter (Beckman/Coulter). All cell counts were performed in triplicate and all experiments were repeated twice. ANOVA with Dunnett’s Multiple Comparison Test was used to determine statistical significance of each infection condition compared to uninfected controls at all timepoints.
